# The effects of psilocybin on cognitive and emotional functions in healthy participants: Results from a phase 1, randomised, placebo-controlled trial involving simultaneous psilocybin administration and preparation

**DOI:** 10.1177/02698811211064720

**Published:** 2022-01-04

**Authors:** James J Rucker, Lindsey Marwood, Riikka-Liisa J Ajantaival, Catherine Bird, Hans Eriksson, John Harrison, Molly Lennard-Jones, Sunil Mistry, Francesco Saldarini, Susan Stansfield, Sara J Tai, Sam Williams, Neil Weston, Ekaterina Malievskaia, Allan H Young

**Affiliations:** 1 Department of Psychological Medicine, Institute of Psychiatry, Psychology & Neuroscience, King’s College London, London, UK; 2 South London and Maudsley NHS Foundation Trust, London, UK; 3 COMPASS Pathways PLC, London, UK; 4 Clinical Research Institute, Helsinki University Central Hospital, Helsinki, Finland; 5 Alzheimer’s Center, AUmc, Amsterdam, The Netherlands; 6 Metis Cognition Ltd., Kilmington Common, UK; 7 Division of Psychology & Mental Health, The University of Manchester, Manchester, UK

**Keywords:** Psilocybin, cognition, emotional processing, placebo-controlled, randomised clinical trial

## Abstract

**Background:**

Psilocybin, a psychoactive serotonin receptor partial agonist, has been reported to acutely reduce clinical symptoms of depressive disorders. Psilocybin’s effects on cognitive function have not been widely or systematically studied.

**Aim:**

The aim of this study was to explore the safety of simultaneous administration of psilocybin to healthy participants in the largest randomised controlled trial of psilocybin to date. Primary and secondary endpoints assessed the short- and longer-term change in cognitive functioning, as assessed by a Cambridge Neuropsychological Test Automated Battery (CANTAB) Panel, and emotional processing scales. Safety was assessed via endpoints which included cognitive function, assessed by CANTAB global composite score, and treatment-emergent adverse event (TEAE) monitoring.

**Methods:**

In this phase 1, randomised, double-blind, placebo-controlled study, healthy participants (*n* = 89; mean age 36.1 years; 41 females, 48 males) were randomised to receive a single oral dose of 10 or 25 mg psilocybin, or placebo, administered simultaneously to up to six participants, with one-to-one psychological support – each participant having an assigned, dedicated therapist available throughout the session.

**Results:**

In total, 511 TEAEs were reported, with a median duration of 1.0 day; 67% of all TEAEs started and resolved on the day of administration. There were no serious TEAEs, and none led to study withdrawal. There were no clinically relevant between-group differences in CANTAB global composite score, CANTAB cognitive domain scores, or emotional processing scale scores.

**Conclusions:**

These results indicate that 10 mg and 25 mg doses of psilocybin were generally well tolerated when given to up to six participants simultaneously and did not have any detrimental short- or long-term effects on cognitive functioning or emotional processing.

**Clinical Trial Registration:**

EudraCT (https://www.clinicaltrialsregister.eu/) number: 2018-000978-30.

## Introduction

Psilocybin (4-phosphoryloxy-N,N-dimethyltryptamine) is a naturally occurring psychoactive alkaloid, first isolated from Psilocybe mushrooms (Passie et al., 2002). Alongside other tryptamines, including dimethyltryptamine (DMT) and ergolines such as lysergic acid diethylamide (LSD), psilocybin is a partial agonist of serotonin (5-hydroxytryptamine; 5-HT) receptors (Halberstadt and Geyer, 2011) and belongs to a class of drugs called ‘psychedelics’ (Carhart-Harris et al., 2016).

Psilocybe mushrooms, and other psychedelic plants such as peyote and ayahuasca, have been used by Central and South American native groups in sacred spiritual and healing ceremonies for thousands of years (Clarke, 2007). In the West, psilocybin has been used in psychiatric research and in psychodynamic-orientated psychotherapy from the early to mid-1960s, after it was first isolated and later synthesised by Albert Hofmann in 1957 and 1958, respectively (Hofmann et al., 1958, 1959). This research on the effects of psilocybin largely halted when it became a Schedule 1 substance due to international legal controls (Rucker et al., 2020), before a resurgence in the mid-1990s (Passie, 2005; Passie et al., 2002).

Psilocybin produces a non-ordinary state of consciousness characterised by changes in emotional state and perception, including experiences of self, space and time (Hasler et al., 2004; Kraehenmann et al., 2015; Vollenweider et al., 1998, 2007). Previous reports suggest that the psychoactive effects of psilocybin are mainly attributable to partial agonism of the 5HT_2A_ receptor subtype (Halberstadt and Geyer, 2011; Madsen et al., 2019; Passie et al., 2002; Vollenweider et al., 1998).

Pilot studies have reported on the efficacy of psilocybin in, for example, treatment-resistant depression (Carhart-Harris et al., 2016, 2018), major depressive disorder (Davis et al., 2021), terminal-cancer–related anxiety (Griffiths et al., 2016; Grob et al., 2011; Ross et al., 2016), obsessive-compulsive disorder (OCD) (Moreno et al., 2006) and alcohol and nicotine dependence (Bogenschutz et al., 2015; Johnson et al., 2014). All reported encouraging efficacy findings, with minimal adverse events (AEs), although these results should be interpreted with caution given that some studies were not specifically designed to robustly test psilocybin’s efficacy (e.g. some had open-label designs or lacked a placebo control arm) and all were limited by small sample sizes. Results from a systematic review of studies conducted prior to psilocybin prohibition in the 1970s were also encouraging (Rucker et al., 2016).

Neural correlates of cognitive and emotional processing are considered important therapeutic targets in the development of novel treatments for treatment-resistant depression and other mental disorders. Recent studies demonstrated that psilocybin and similar psychedelics modulate neural circuits implicated in affective disorders, with potential to reduce clinical symptoms of depression (Carhart-Harris et al., 2012, 2016, 2018).

Social cognitive functioning is an influential factor in the development, progression and treatment of many mental health problems. Deficits in social cognition represent key characteristics of many psychiatric and neurological disorders (Cotter et al., 2018) and may compromise real-world functioning across various settings, including work and independent living (Arioli et al., 2018; Jones et al., 2015). Current interventions for psychiatric disorders include psychosocial techniques, such as psychotherapies and occupational therapy and/or social environment and role interventions, as well as pharmacological treatments. Although these interventions are successful for some, they fail others and are only partially successful in many. Lack of an adequate breadth of interventions for mental health problems, combined with an increasingly overt impact, highlights an urgent need for alternative approaches (NHS Digital, 2014). Given this unmet need for improved treatment of social and emotional functioning deficits, a better understanding of the short- and long-term effects of psilocybin on emotional processing and social cognition is required.

The serotonin system has been reported to represent a promising target for pharmacological modulation of social and emotional functioning and has been implicated in the pathophysiology of various mental health problems (Ciranna, 2006; Švob Štrac et al., 2016). Increasing serotonin levels in healthy participants by administering a selective serotonin reuptake inhibitor promoted prosocial behaviour in one study (Crockett et al., 2010) and reduced processing of negative emotional stimuli in another (Harmer et al., 2004); however, since serotonin reuptake inhibitors block 5-HT uptake, such studies do not provide information on specific 5-HT-receptor functioning in social and emotional functioning. Since psilocybin is a preferential partial agonist of the 5-HT_2A_ and 5-HT_1A_ receptors, it is well-suited for investigation of the relative contributions of these subtypes to aspects of emotional processing. In healthy participants, 5-HT_2A_/_1A_-receptor stimulation following psilocybin administration acutely enhanced mood and empathy (Mason et al., 2019; Pokorny et al., 2017), prosocial behaviour (Gabay et al., 2018) and attenuated processing of negative facial expressions and social pain (Bernasconi et al., 2014; Kometer et al., 2012; Preller et al., 2016). While these acute effects of psilocybin indicate that serotonin receptor stimulation is a promising target for improvement of emotional processing, investigation of the longer-term effects is of clinical relevance, as it is unclear whether psilocybin’s prosocial effects persist post-acutely. Furthermore, it has not yet been investigated whether the beneficial effects of psilocybin may also benefit general cognitive abilities. Given the potential for psilocybin as a treatment for a range of conditions, studying its effects on cognition is important.

This study aimed to evaluate the safety and feasibility of simultaneous administration of single doses of either 10 or 25 mg doses of psilocybin compared with placebo in healthy participants, administered in a supervised setting with one-to-one psychological support, and to assess the short- and long-term effects of psilocybin on key domains of cognitive functioning. To our knowledge, this represents the largest randomised controlled trial of psilocybin to date. It is important to study the safety and feasibility of this model of simultaneous administration as it could be a more effective, time- and cost-efficient model of treatment delivery, meaning more patients could potentially benefit from this treatment, if approved. While the psychological support in psilocybin therapy delivered before and after psilocybin administration has been administered in group settings in another recent study by Anderson et al. (2020), the actual administration of psilocybin has only been given on an individual basis in modern studies, to date. Prior to the implementation of legal constraints surrounding psychedelics and subsequent assignment of psilocybin and LSD as Schedule 1 substances, several studies did administer psychedelics and/or psychedelic therapy sessions in a group setting (e.g. Harman et al., 1966; Leary et al., 1963; Pahnke, 1963). This study is the first study since the revival of psychedelic research, to our knowledge, to administer psilocybin to participants simultaneously.

## Materials and methods

### Study design

This was a phase 1, randomised, double-blind, placebo-controlled, between-groups study to evaluate the effects of single 10 and 25 mg doses of psilocybin compared with placebo in healthy participants, conducted at the Institute of Psychiatry, Psychology and Neuroscience, King’s College London, London, UK, sponsored by COMPASS Pathways (EudraCT number: 2018-000978-30).

### Participants

The study recruited healthy participants (male or female; aged 18−65 years at screening) with no psilocybin experience within 1 year prior to enrolment; the study aimed to recruit up to 90 participants who met eligibility criteria at the screening visit (visit 1; day -56 to day -2). Participants were recruited via online advertisements and word of mouth. Inclusion criteria, psychiatric exclusion criteria (including current/history of psychiatric disorders, current/recent psychiatric medications, or other psilocybin-incompatible psychiatric or psychological conditions, as judged by the investigator), and medical exclusion criteria are listed in the Supplementary Methods.

### Procedures

Participants were followed up for 12 weeks after study drug administration; the visit schedule is presented in Supplementary Figure S1a. Eligibility screening (visit 1) involved medical and psychiatric history assessment; administration of the Mini International Neuropsychiatric Interview v7.0.2 (Sheehan et al., 1998), the MacLean Screening Instrument for Borderline Personality Disorder (Zanarini et al., 2003) and the Sheehan Suicidality Tracking Scale (SSTS (Coric et al., 2009); administered at screening, baseline and all post-baseline visits except visit 7); physical examination; assessment of vital signs, body weight, height and body mass index; 12-lead electrocardiogram; clinical laboratory tests; urine drug screen; urine pregnancy test; documentation of contraceptive method; review of prior and concomitant medications; and recording of AEs. On the day before study drug administration (baseline; day -1; visit 2), participants underwent assessments of cognitive functioning and emotional processing including social cognition tasks, vital signs, urine drug screen, review of prior/concomitant medications and recording of AEs (Supplementary Figure S1b). Participants were stratified by sex and age (18–35 years old; >35 years old) and randomised in a 1:1:1 ratio using blocked randomisation (Supplementary Methods) to 10 mg psilocybin, 25 mg psilocybin, or placebo, to be administered orally. During this visit, participants attended a 2-h group preparatory session (involving all participants to be dosed the following day, a lead therapist and assisting therapists) and had a short (5–15 min) individual discussion with their assigned assisting therapist. Details of therapist training and qualifications are provided elsewhere (Tai et al., 2021).

On day 1, participants were instructed to eat a light breakfast ⩾ 2 h before arriving at the clinic; prior to receiving study drug, participants underwent the SSTS, evaluation of vital signs and review of concomitant medications and AEs (Supplementary Figure S1b). The study drug was administered simultaneously to up to six participants as a single 5-capsule oral dose (psilocybin 10 mg: 2 × 5 mg psilocybin capsules plus 3 × placebo capsules; psilocybin 25 mg: 5 × 5 mg psilocybin capsules; placebo: 5 × placebo capsules). COMP360 psilocybin was administered in this study, which is COMPASS Pathways’ proprietary pharmaceutical-grade synthetic psilocybin formulation that has been optimised for stability and purity. Participants were randomised on an individual basis and therefore participants in the same dosing session could be allocated to different treatment groups.

Administration sessions generally lasted ~6–8 h, with psychological support available throughout from specially trained assisting therapists, who were supervised by a lead therapist. The first effects of psilocybin are seen about 20–30 min after administration, are most intense in the first 90–120 min and then gradually subside, typically resolving in ~5−6 h after administration (Carhart-Harris et al., 2016). The simultaneous dosing involved each participant having a private space, for example, a bed separated by curtains within the same room, so they could focus on their own experience with minimal distractions, especially as participants were encouraged to wear eyeshades, earplugs and/or earphones for the duration of the administration session. Participants communicated only with their therapists during the administration sessions.

After at least 6 h, once the effects of the study drug had mostly subsided, participants were assessed for safety by the clinical judgement of therapists and a psychiatrist, AEs were collected, and participants were asked to complete the Positive and Negative Affect Schedule (PANAS) (Watson and Clark, 1994; Watson et al., 1988).

Participants were subsequently discharged and asked to return the following day (day 1; visit 4) for safety assessments and a discussion with their allocated therapist about the subjective experience during the administration session (integration session).

### Funding and ethical approval

All participants signed an informed consent form prior to eligibility screening. The study was conducted in accordance with the International Conference on Harmonisation guidelines for Good Clinical Practice, the regulations on electronic records and electronic signature, and the most recent guidelines of the Declaration of Helsinki, and was approved by the London-Brent Research Ethics Committee (reference number: 18/LO/0731). The study was funded and sponsored by COMPASS Pathways, London, UK.

### Study endpoints

Safety endpoints included cognitive function, suicidality (as measured by the SSTS; a higher score indicates greater suicidality); AEs and serious AEs; vital signs; and clinical laboratory tests (basic chemistry test). Cognitive function was measured by the Cambridge Neuropsychological Test Automated Battery (CANTAB) global composite score change from baseline (day -1) to day 8 and 29, calculated from Z scores for each CANTAB measure (Paired Associates Learning-Total Errors Adjusted (PAL-TEA; a measure of episodic memory); Spatial Working Memory-Between Errors (SWM-BE; working memory); SWM-Strategy (SWM-S; executive function and planning); and Rapid Visual Information Processing A-prime (RVP-A’; sustained attention); a higher CANTAB global composite score indicates better cognitive function). Each AE reported by a participant was assessed for its relationship to the study drug by the blinded study investigator reporting the event with the following criteria: related, possibly related, or not related.

The primary efficacy endpoints were short-term change from baseline (day -1) to day 8 in cognitive measures of attention, spatial and working memory and executive function (Sahakian and Owen, 1992); short-term change from baseline to day 8 in social cognition, emotional processing scales (comprising the Pictorial Empathy Test (PET); Reading the Mind in the Eyes Test (RMET); Scale of Social Responsibility (SSR); Social Value Orientation (SVO); and Toronto Empathy Questionnaire (TEQ) (Baron-Cohen et al., 2001; Gough et al., 1952; Lindeman et al., 2018; Murphy et al., 2011; Spreng et al., 2009)); change from baseline to day 29 in the individual cognitive functioning assessments previously outlined; and long-term change from baseline to day 85 in social cognition, emotional processing scales.

Secondary efficacy endpoints included dose-related differences in the cognitive effects of psilocybin at baseline, day 8 and day 29, measured by the CANTAB assessments RVP-Aʹ, SWM-BE, SWM-S, PAL-TEA; dose-related differences in the psychological effects of psilocybin at baseline, day 8 and day 85, measured by the social cognition, emotional processing scales; differences in the cognitive effects of psilocybin between psilocybin-naïve and -experienced participants at baseline, day 8 and day 29, measured by the CANTAB global composite score; and differences in PANAS after study drug administration on day 1.

### Statistical analyses

The Safety Population comprised all randomised participants who received study drug and was used for all summaries of participant accountability, demographic and baseline data, and safety information, including AE incidence and cognition endpoint analyses. The modified Intent-to-Treat (mITT) Population comprised all participants in the Safety Population who had at least one post-dose assessment. This population was used for emotional processing endpoint analyses. The Per-Protocol (PP) Population comprised all participants in the Safety Population who did not have a major protocol deviation that was thought to significantly affect the integrity of the participant’s data. This population was used for the CANTAB primary endpoint analyses.

A sample size of up to 90 participants (30 per treatment group) was chosen to allow reasonable assessment of each of the planned endpoints. This study was exploratory and therefore not adequately powered to detect statistical significance; as such *p* values are not reported. The effects of psilocybin on each outcome variable collected at more than one time point post-baseline were evaluated as change from baseline scores (observed case) using a mixed-model for repeated measures (MMRM) analysis with factors for administration group, former psilocybin experience (FPE) [emotional processing endpoints only], visit, and treatment-by-visit interaction as fixed effects, and baseline value as a covariate. An unstructured variance covariance matrix was used to model the within-patient errors, and the Kenward and Roger method was used for calculating the denominator degrees of freedom for tests of fixed effects. The MMRM method utilises the observed data efficiently, handling missing data automatically, based on the assumption that data are missing at random, thus minimising bias in the estimates of treatment effect. The PANAS, which was assessed at baseline and day of administration, was analysed using an Analysis of Covariance (ANCOVA) model with change from baseline as the dependent variable; the model included fixed effects for study drug and FPE, and baseline score as a covariate. To investigate the impact of prior psilocybin use (yes/no) on the CANTAB endpoints (safety-related), the MMRM model above was extended to include the main effect for FPE and its interactions with study drug and time.

Safety analyses were performed on evaluation of CANTAB global composite score, AEs (coded by Medical Dictionary for Regulatory Activities (MedDRA) Version 21.0 preferred term and summarised by treatment group, severity and relationship to study drug determined by the investigator reporting the AE), vital signs and clinical laboratory assessments. Treatment-emergent AEs (TEAEs) were defined as any AEs with an onset on or after the dose of study drug, or any pre-existing condition that worsened on or after the dose of study drug.

At screening, participants underwent CANTAB assessments as part of a familiarisation session; this was included in the descriptive statistics but was excluded from statistical modelling.

All analyses were performed using statistical software SAS^®^ (SAS Institute Inc., Cary, NC, USA).

## Results

### Participants

The study randomised 89 healthy participants (mean age, 36.1 years; 41 females, 48 males); participant demographics and disposition are summarised in Table 1 and Supplementary Figure S2, respectively. Of these, 30 participants were randomised to receive 25 mg psilocybin, 30–10 mg psilocybin and 29 to placebo; 33 (37.1%) participants had prior psilocybin experience. Twenty-five administration sessions were completed, with up to six participants dosed simultaneously per session (there were 2 sessions with one participant each, 3 with two, 5 with three, 11 with four, 2 with five and 2 with six). All participants randomised to both psilocybin arms completed the study; four (13.8%) placebo-treated participants did not complete all study visits (three were lost to follow-up; one was withdrawn following a protocol deviation). The first participant’s first visit date was August 17, 2018, and the last participant’s last visit date was July 19, 2019.

**Table 1.** Participant demographics (safety population).

Parameter	Psilocybin 25 mg (*N* = 30)	Psilocybin 10 mg (*N* = 30)	Placebo (*N* = 29)	Overall (*N* = 89)
Gender, n (%)				
Male	16 (53.3)	16 (53.3)	16 (55.2)	48 (53.9)
Female	14 (46.7)	14 (46.7)	13 (44.8)	41 (46.1)
Race, n (%)				
White	25 (83.3)	27 (90.0)	20 (69.0)	72 (80.9)
Black	0	0	1 (3.4)	1 (1.1)
Asian	2 (6.7)	1 (3.3)	3 (10.3)	6 (6.7)
Mixed	2 (6.7)	1 (3.3)	1 (3.4)	4 (4.5)
Other	1 (3.3)	1 (3.3)	4 (13.8)	6 (6.7)
Age at time of consent, years				
Mean (SD)	36.6 (10.29)	36.1 (9.25)	35.6 (7.69)	36.1 (9.06)
BMI, kg/m^2^				
Mean (SD)	22.9 (3.75)	23.0 (2.90)	23.7 (3.20)	23.2 (3.29)
Educational level, n (%)				
No formal qualifications	0	0	0	0
GCSE/GCE/O level	0	0	0	0
A level/NVQ	2 (6.7)	1 (3.3)	0	3 (3.4)
Undergraduate/higher national diploma	9 (30.0)	11 (36.7)	10 (34.5)	30 (33.7)
Master’s or postgraduate diploma	16 (53.3)	16 (53.3)	15 (51.7)	47 (52.8)
PhD	3 (10.0)	2 (6.7)	4 (13.8)	9 (10.1)
Prior psilocybin experience, n (%)				
Yes	11 (36.7)	15 (50.0)	7 (24.1)	33 (37.1)
No	19 (63.3)	15 (50.0)	22 (75.9)	56 (62.9)

BMI: body mass index; GC(S)E: general certificate of (secondary) education; NVQ: national vocational qualification.

### Safety outcomes

#### Adverse events

In total, 511 TEAEs were reported during the 12-week study: 217 in the 25 mg psilocybin arm (reported by 29 (96.7%) participants), 203 in the 10 mg psilocybin arm (reported by 29 (96.7%) participants) and 91 in the placebo arm (reported by 26 (89.7%) participants). Of these, 208, 188 and 77 were deemed by the investigator to be potentially related to study treatment in the 25 mg, 10 mg and placebo arms, respectively. The most frequently reported TEAEs (ordered according to incidence in the 25 mg arm) and a summary of predefined TEAEs of special interest are presented in Table 2. There were no serious TEAEs and no AEs led to withdrawal from the study.

**Table 2.** Most frequently reported TEAEs (occurring in >15% of participants in any treatment arm and ordered according to incidence in the 25 mg psilocybin arm) and summary of TEAEs of special interest (Safety Population).

	Psilocybin 25 mg (*N* = 30)		Psilocybin 10 mg (*N* = 30)		Placebo (*N* = 29)	
	*n* (%)	Events	*n* (%)	Events	*n* (%)	Events
Most frequently reported TEAE (MedDRA Preferred Term)						
Hallucination, visual	21 (70.0)	22	18 (60.0)	20	2 (6.9)	2
Illusion	18 (60.0)	26	19 (63.3)	25	4 (13.8)	5
Mood altered	15 (50.0)	25	13 (43.3)	23	6 (20.7)	9
Headache	15 (50.0)	16	9 (30.0)	12	5 (17.2)	5
Fatigue	8 (26.7)	8	9 (30.0)	10	3 (10.3)	3
Euphoric mood	7 (23.3)	8	7 (23.3)	7	0	0
Tension headache	6 (20.0)	6	3 (10.0)	3	3 (10.3)	3
Time perception altered	6 (20.0)	6	2 (6.7)	2	3 (10.3)	3
Emotional disorder	5 (16.7)	6	2 (6.7)	2	0	0
Somatic hallucination	5 (16.7)	6	8 (26.7)	8	4 (13.8)	5
Affect lability	3 (10.0)	3	5 (16.7)	5	1 (3.4)	1
TEAEs of special interest (MedDRA System Organ Class/Preferred Term)						
Any TEAE of special interest	26 (86.7)	80	25 (83.3)	81	10 (34.5)	19
Nervous system disorders	0	0	2 (6.7)	2	0	0
Memory impairment	0	0	1 (3.3)	1	0	0
Psychomotor skills impaired	0	0	1 (3.3)	1	0	0
Psychiatric disorders	26 (86.7)	80	25 (83.3)	79	10 (34.5)	19
Affect lability	3 (10.0)	3	5 (16.7)	5	1 (3.4)	1
Change in sustained attention	0	0	2 (6.7)	2	0	0
Depressed mood	2 (6.7)	2	1 (3.3)	1	1 (3.4)	1
Dissociative identity disorder	2 (6.7)	2	1 (3.3)	2	0	0
Euphoric mood	7 (23.3)	8	7 (23.3)	7	0	0
Hallucination^a^	2 (6.7)	2	3 (10.0)	3	0	0
Hallucination, auditory	4 (13.3)	4	4 (13.3)	4	1 (3.4)	1
Hallucination, gustatory	0	0	1 (3.3)	1	0	0
Hallucination, olfactory	1 (3.3)	1	1 (3.3)	1	0	0
Hallucination, tactile	4 (13.3)	4	2 (6.7)	2	0	0
Hallucination, visual	21 (70.0)	22	18 (60.0)	20	2 (6.9)	2
Mood altered	15 (50.0)	25	13 (43.3)	23	6 (20.7)	9
Somatic hallucination	5 (16.7)	6	8 (26.7)	8	4 (13.8)	5
Substance-induced psychotic disorder	1 (3.3)^b^	1	0	0	0	0

TEAEs were coded post hoc to MedDRA Version 21.0 Preferred Terms. TEAE: treatment-emergent adverse event; MedDRA: Medical Dictionary for Regulatory Activities.

a All TEAEs coded to the MedDRA preferred term ‘Hallucination’ were described as ‘kinaesthetic hallucinations’.

Four participants reported AEs of anxiety on the day of study drug administration (25 mg psilocybin, *n* = 2 (6.7%); 10 mg psilocybin, *n* = 1 (3.3%); placebo, *n* = 1 (3.4%)) In total, 57 AEs of ‘mood altered’ were reported (mood-related AEs were grouped into this MedDRA preferred term post hoc, while retaining the AE description originally reported by the participant/investigator); of these, two were negative alterations in mood, one in the 10 mg psilocybin arm (‘feeling more moody or sensitive’, which started on day 3 and lasted for 9 days) and one in the placebo arm (‘negative mood’, which started and resolved on day 1). The remaining ‘mood altered’ AEs, which included introspection, reflection and sense of oneness, are summarised in Supplementary Table S1.

Psilocybin induced expected, transient psychedelic experiences. These included 86 reports of hallucination, 57 of mood altered, 56 of illusion, 15 of euphoric mood and 11 of time perception altered. Across all AEs reported in all treatment arms throughout the 12 week study, 67% started and resolved on the administration day; the median duration of AEs was 1.0 day. When selecting only those AEs likely to be psychedelic in nature (according to the AE MedDRA preferred term), determined by post hoc adjudication by four investigators (54% of all AEs), 255 started on the administration day, 235 (92%) of which were resolved that day.

An AE of substance induced psychotic disorder was reported for a participant who became behaviourally disinhibited during the acute drug experience. After a medical assessment, 2.5 mg oromucosal midazolam was administered. The participant recovered with no sequelae and was discharged 11 h after receiving the study intervention. This event was not considered to be an SAE, and no clinically significant ongoing effects were noted at follow-up.

#### Clinical Laboratory Assessment and Vital signs

There were four clinically significant clinical laboratory assessment findings, which were recorded as AEs (none of which prevented the participant from entering or continuing in the study). Two occurred in the blood test at screening, two in the blood test on the day after administration. There were no clinically significant findings in vital signs.

#### Suicidality

At baseline, all participants recorded an SSTS score of 0. During follow-up, no participants in the 25 mg psilocybin arm recorded an SSTS score **>** 0; however, one participant in the 10 mg psilocybin arm recorded a highest score of 1 during follow-up (reported at the day 29 visit). This participant reported a TEAE of suicidal thoughts (one event), which started and resolved on day 19, was mild in severity and was deemed by the investigator to be possibly related to study drug. In addition, one participant in the placebo arm reported two TEAEs of suicidal ideation (one started on day 4 and lasted for 5 days; one started on day 18 and lasted for 17 days) and two TEAEs of suicidal thoughts (one started and resolved on day 79; one started and resolved on day 91). All four events were moderate in severity and considered possibly related to study drug.

### Cognitive and emotional processing outcomes

#### Cognitive functioning

**Figure 1.**
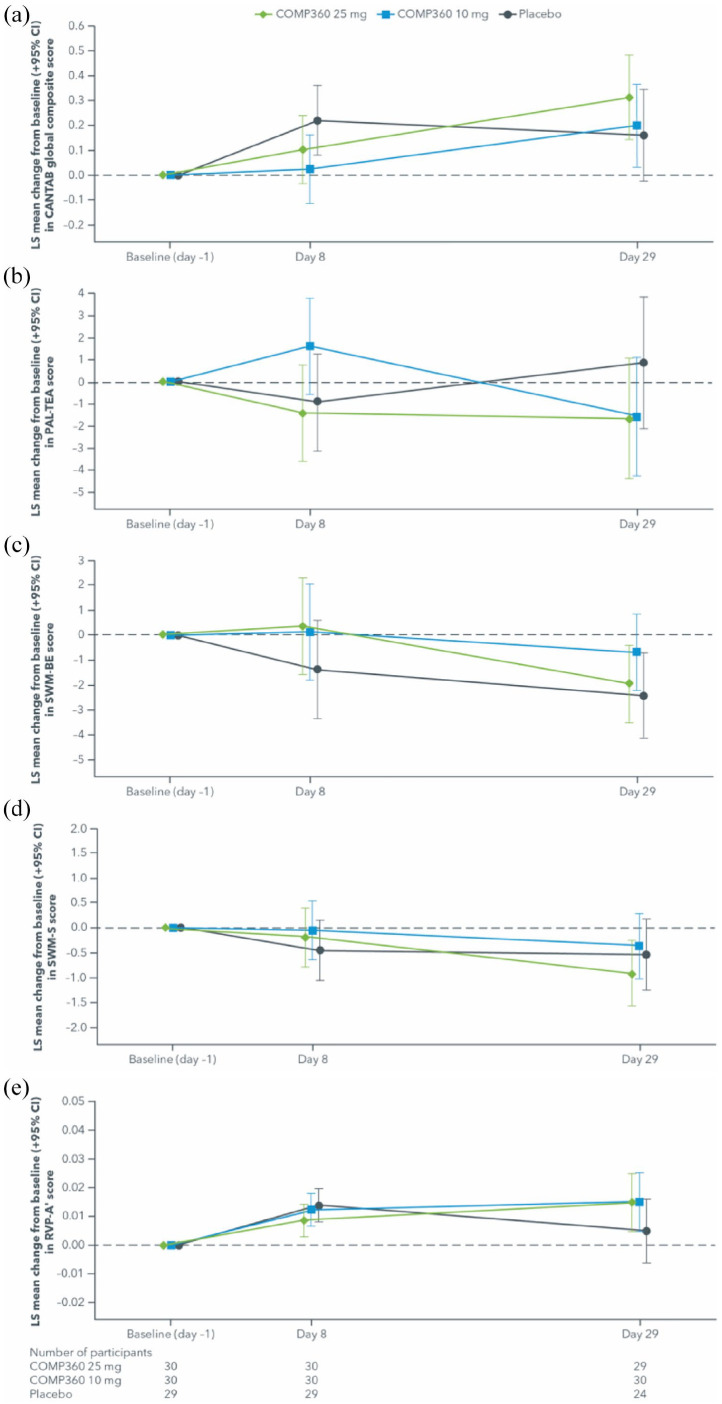
Mixed-model analysis of change from baseline in CANTAB outcome measures (Safety Population) in: a) CANTAB global composite score, b) PAL-TEA score, c) SWM-BE score, d) SWM-S score, e) RVP-A’ score. CANTAB: Cambridge Neuropsychological Test Automated Battery; LS: least squares; PAL-TEA: Paired Associates Learning-Total Errors Adjusted; RVP-A’: Rapid Visual Information Processing A-prime; SWM-BE: Spatial Working Memory-Between Errors; SWM-S: Spatial Working Memory-Strategy.

Mixed-model analysis of change from baseline in CANTAB outcomes measures by dose (Safety Population) are presented in Table 3 and Figure 1(a) to (e).

**Table 3.** Mixed-model analysis of change from baseline in CANTAB outcome measures (safety population).

	LS mean (SE) change from baseline in score		LS mean (95% CI) difference from placebo	
	Day 8	Day 29	Day 8	Day 29
CANTAB global composite				
Placebo	0.2197 (0.07017)	0.1617 (0.09272)	—	—
Psilocybin 10 mg	0.0237 (0.06899)	0.1981 (0.08376)	−0.1960 (−0.39172, −0.00024)	0.0364 (−0.21234, 0.28505)
Psilocybin 25 mg	0.1030 (0.06898)	0.3136 (0.08501)	−0.1167 (−0.31228, 0.07898)	0.1519 (−0.09846, 0.40219)
PAL-TEA				
Placebo	−0.9 (1.11)	0.9 (1.50)	—	—
Psilocybin 10 mg	1.6 (1.09)	−1.6 (1.35)	2.5 (−0.57, 5.65)	−2.4 (−6.48, 1.59)
Psilocybin 25 mg	−1.4 (1.09)	−1.7 (1.37)	−0.5 (−3.60, 2.60)	−2.5 (−6.59, 1.52)
SWM-BE				
Placebo	−1.4 (0.99)	−2.4 (0.85)	—	—
Psilocybin 10 mg	0.1 (0.98)	−0.7 (0.77)	1.5 (−1.27, 4.27)	1.7 (−0.56, 4.02)
Psilocybin 25 mg	0.3 (0.97)	−2.0 (0.78)	1.7 (−1.02, 4.51)	0.5 (−1.83, 2.77)
SWM-S				
Placebo	−0.5 (0.31)	−0.5 (0.36)	—	—
Psilocybin 10 mg	−0.1 (0.30)	−0.4 (0.33)	0.4 (−0.46, 1.26)	0.2 (−0.78, 1.16)
Psilocybin 25 mg	−0.2 (0.30)	−0.9 (0.33)	0.3 (−0.59, 1.12)	−0.4 (−1.35, 0.60)
RVP-A’				
Placebo	0.0139 (0.00293)	0.0049 (0.00558)	—	—
Psilocybin 10 mg	0.0122 (0.00288)	0.0152 (0.00502)	−0.0017 (−0.00984, 0.00651)	0.0103 (−0.00464, 0.02524)
Psilocybin 25 mg	0.0086 (0.00288)	0.0148 (0.00510)	−0.0053 (−0.01348, 0.00286)	0.0099 (−0.00519, 0.02490)

Baseline is defined as the last measurement obtained prior to study drug administration.

The treatment effect (i.e. the difference between LS means for each treatment pair) is obtained from a mixed-model for repeated measures analysis with change from baseline score as the dependent variable. The model includes fixed effects for treatment, visit and treatment-by-visit interaction, with visit as the repeating factor, participant as a random effect and baseline score as a covariate. For PAL-TEA, SWM-BE and SWM-S, lower scores denote better performance. For RVP-A’ and CANTAB composite, higher scores denote better performance. CANTAB: Cambridge Neuropsychological Test Automated Battery; LS: least squares; CI confidence interval; PAL-TEA: Paired Associates Learning-Total Errors Adjusted; SWM-BE: Spatial Working Memory-Between Errors; SWM-S: Spatial Working Memory-Strategy; RVP-A’: Rapid Visual Information Processing A-prime.

For RVP-A’, a measure of sustained attention, there were trends indicating better performance on average for 10 mg and 25 mg psilocybin by day 29 compared with baseline, but no difference was observed for 10 mg and 25 mg psilocybin when compared with placebo, nor between 25 mg and 10 mg psilocybin. For SWM-BE, a measure of working memory, and SWM-S, a measure of executive function and planning, there were trends indicating better performance on average for 25 mg psilocybin (and placebo for SWM-BE only) by day 29 compared with baseline, but no difference was observed for 10 mg and 25 mg psilocybin when compared with placebo, nor between 25 mg and 10 mg psilocybin. For PAL-TEA, a measure of episodic memory, there was no difference for any of the groups at day 29 compared with baseline, nor were any differences observed between the groups.

For the global composite (safety outcome), higher scores indicate better performance. Overall, there was an increasing trend in score for the 10 mg and 25 mg psilocybin doses by day 29 compared with baseline. But no difference was observed for 10 mg and 25 mg psilocybin when compared with placebo, and also between 25 mg and 10 mg psilocybin by day 29. On the CANTAB composite score, performance was worse than placebo for the 10 mg psilocybin group at day 8. However, this result is due in part to the larger improvement in performance from baseline by the placebo group at day 8. For the 10 mg psilocybin group, performance increased again by day 29 to a level similar to placebo suggesting no adverse effects of the 10 mg psilocybin dose compared with placebo.

When comparing findings on the aforementioned CANTAB assessments (RVP-A’, SWM-BE, SWM-S, PAL-TEA) between psilocybin-naïve and psilocybin-experienced participants and in the Per-Protocol population (which excluded one participant as their baseline CANTAB assessment was not completed until after the administration session), the results suggested there was no difference of interest and the results were similar to what was observed in the overall analysis in the Safety Population. It should be noted that there were small numbers of participants in each subgroup, as well as an imbalance in the number of participants who were psilocybin-naïve and experienced in the 25 mg psilocybin arm and placebo.

#### Social cognition and emotional processing

There was no difference between either psilocybin group and placebo in any social cognition and emotional processing scale (PET, RMET, SSR, SVO, or TEQ) scores at day 8 or day 85, relative to baseline (Table 4).

**Table 4.** Mixed-model analysis of change from baseline in social cognition scales (Modified Intent-to-Treat Population).

	LS mean (SE) change from baseline in score		LS mean (95% CI) difference from placebo	
	Day 8	Day 85	Day 8	Day 85
PET				
Placebo	−0.1 (0.10)	−0.2 (0.10)	–	–
Psilocybin 10 mg	−0.3 (0.09)	−0.3 (0.09)	−0.1 (−0.39, 0.15)	−0.1 (−0.39, 0.15)
Psilocybin 25 mg	0.0 (0.09)	−0.1 (0.09)	0.2 (−0.11, 0.42)	0.1 (−0.12, 0.41)
RMET				
Placebo	0.3 (0.59)	−0.1 (0.66)	–	–
Psilocybin 10 mg	0.4 (0.53)	0.1 (0.57)	0.1 (−1.43, 1.69)	0.2 (−1.53, 1.95)
Psilocybin 25 mg	0.4 (0.55)	0.4 (0.61)	0.2 (−1.44, 1.74)	0.5 (−1.29, 2.28)
SSR Global				
Placebo	−3.2 (1.23)	−2.1 (1.20)	–	–
Psilocybin 10 mg	−2.0 (1.12)	−1.8 (1.00)	1.2 (−2.16, 4.56)	0.3 (−2.83, 3.49)
Psilocybin 25 mg	−0.3 (1.17)	0.1 (1.04)	2.8 (−0.50, 6.18)	2.2 (−0.94, 5.29)
SSR Fulfilling Expectations				
Placebo	−0.2 (0.07)	−0.1 (0.07)	–	–
Psilocybin 10 mg	−0.1 (0.07)	−0.2 (0.06)	0.1 (−0.14, 0.26)	−0.1 (−0.32, 0.08)
Psilocybin 25 mg	0.0 (0.07)	0.0 (0.07)	0.2 (−0.04, 0.35)	0.0 (−0.16, 0.23)
SSR Compliance Social Rules				
Placebo	−0.1 (0.08)	−0.1 (0.08)	–	–
Psilocybin 10 mg	−0.1 (0.07)	−0.1 (0.07)	0.0 (−0.19, 0.22)	0.0 (−0.22, 0.22)
Psilocybin 25 mg	0.0 (0.07)	0.0 (0.07)	0.1 (−0.07, 0.34)	0.1 (−0.08, 0.35)
SVO Angle				
Placebo	−1.6 (1.29)	−3.3 (1.37)	–	–
Psilocybin 10 mg	1.8 (1.16)	0.1 (1.16)	3.4 (−0.05, 6.85)	3.4 (−0.16, 6.97)
Psilocybin 25 mg	0.5 (1.20)	−0.9 (1.24)	2.1 (−1.32, 5.58)	2.4 (−1.16, 6.06)
SVO Type				
Placebo	0.0 (0.07)	0.0 (0.06)	–	–
Psilocybin 10 mg	0.1 (0.06)	0.1 (0.05)	0.1 (−0.07, 0.28)	0.1 (−0.06, 0.28)
Psilocybin 25 mg	0.1 (0.06)	0.0 (0.06)	0.1 (−0.11, 0.24)	0.1 (−0.10, 0.24)
TEQ				
Placebo	−0.1 (0.09)	−0.2 (0.09)	–	–
Psilocybin 10 mg	−0.1 (0.08)	−0.1 (0.08)	0.1 (−0.16, 0.31)	0.1 (−0.15, 0.34)
Psilocybin 25 mg	0.0 (0.08)	0.0 (0.08)	0.2 (−0.08, 0.38)	0.1 (−0.13, 0.36)

Baseline is defined as the last measurement obtained prior to study drug administration.

The treatment effect (i.e. the difference between LS means for each treatment pair) is obtained from a mixed-model for repeated measures analysis with change from baseline score as the dependent variable. The model includes fixed effects for treatment, Former Psilocybin Experience, visit and treatment-by-visit interaction, with visit as the repeating factor, participant as a random effect and baseline score as a covariate. LS: least squares; CI: confidence interval; PET: Pictorial Empathy Test; RMET: Reading the Mind in the Eyes Test; SSR: Scale of Social Responsibility; SVO: Social Value Orientation; TEQ: Toronto Empathy Questionnaire.

#### PANAS

The placebo group showed a trend for a reduction in positive affect score from baseline to post-dose (least squares (LS) mean change = -5.0), which was not observed in either psilocybin group (Supplementary Table S3). Conversely, the 25 mg psilocybin group showed a trend for an increase in LS mean negative affect score from baseline of 1.3, compared with no change in the 10 mg psilocybin and placebo groups.

## Discussion

This was a phase 1, double-blind, randomised, placebo-controlled study to evaluate the effects of a single dose (10 mg or 25 mg) of psilocybin, with one-to-one support from specially trained therapists, on cognitive functioning and emotional processing in healthy participants. The study demonstrated the safety and feasibility of this model of psilocybin administration, as demonstrated by willingness of participants to undergo simultaneous administration and group preparation sessions.

Psilocybin was generally well tolerated, with no serious TEAEs reported, consistent with findings of previous, smaller studies evaluating the feasibility of psilocybin administration with psychological support in patients with psychiatric disorders (Bogenschutz et al., 2015; Carhart-Harris et al., 2016, 2018; Davis et al., 2021; Griffiths et al., 2016; Grob et al., 2011; Johnson et al., 2014; Moreno et al., 2006; Ross et al., 2016). No TEAEs led to study withdrawal, indicating that psilocybin administered at these doses in a supervised setting was well tolerated among healthy participants. Over two thirds of adverse events both started and resolved on the day of dosing. Both doses of psilocybin elicited expected, transient psychedelic effects, which in previous studies have correlated with antidepressive/anxiolytic efficacy (Carhart-Harris and Goodwin, 2017; Davis et al., 2021; Griffiths et al., 2016; Grob et al., 2011).

Small differences in cognitive outcomes were seen between the groups, but no clinically relevant negative findings were identified. For RVP-A’, SWM-BE, SWM-S and CANTAB global composite, there were trends demonstrating better performance on average in the psilocybin groups by day 29 compared with baseline. The fact that participants were typically highly educated, and the small sample size, could have limited the generalisability of results. These findings warrant further investigation in clinical populations.

In terms of social cognition and emotional processing outcomes, there were no consistent trends to suggest that either psilocybin dose had a short- or long-term effect on any social cognition scale (PET, RMET, SSR, SVO, or TEQ). These findings suggest that psilocybin does not exert any detrimental effect on the social cognition and emotional functions assessed. Future research should evaluate the generalisability of these social cognition findings in clinical populations. Evaluation of PANAS scores measured immediately following study drug administration showed a reduction in positive affect for placebo-treated participants, which was not replicated in either psilocybin dose group, whereas the 25 mg arm showed an increase in negative affect, which was not replicated in the 10 mg or placebo groups.

Taken together, these findings support the exploration of psilocybin for the treatment of psychiatric disorders, including treatment-resistant depression, in a supervised setting with psychological support. Further studies are required to investigate the efficacy and safety of psilocybin in patient populations in this setting.

### Strengths and limitations

This was the first study, to our knowledge, to systematically study the longer-term effects of psilocybin on cognition, and to report a full AE profile in healthy volunteers. This study benefitted from a larger sample size than previous studies of the subjective experience, efficacy and safety of psilocybin (Carhart-Harris et al., 2016, 2018; Griffiths et al., 2008, 2011, 2016); however, our sample size was not powered to detect statistically significant differences in psilocybin efficacy between groups. Instead, this was an exploratory evaluation of the efficacy and safety of psilocybin compared with placebo; caution should therefore be taken when interpreting these results.

Although both 10 mg and 25 mg of psilocybin were generally well tolerated by participants in this study, it is important to consider the possible risks. Previous literature, albeit in very rare cases (Dos Santos et al., 2017; Litjens et al., 2014; Nichols, 2016), has reported a few incidences of serotonin syndrome (Schifano et al., 2021), Hallucinogen Persisting Perception Disorder (HPPD) (Litjens et al., 2014) and substance-related exogenous psychosis (Hendin and Penn, 2021) with psychedelic substances, typically when used recreationally alongside other psychotropic medications. More research including larger, diverse samples are necessary to gain a clearer picture of the acute and longer-term adverse events associated with psilocybin.

The current study measured the short-term effects of psilocybin at day 8, and longer-term effects at day 29 or 85. Future research would benefit from collecting outcomes at later time points post-dosing to gain a broader understanding of more long-term effects, and also acutely, either immediately after or during psilocybin administration.

General population level lifetime use of psilocybin in the UK population is estimated to be approximately 3%, with a slightly higher use of 6% of the population reported in the United States (Hill and Thomas, 2020; Krebs and Johansen, 2013). With thirty-five participants reporting previous use of psilocybin in this study, the proportion of participants with prior experience is greater than expected in the general population, thus additional caution should be taken when generalising these results to other populations. In many previous psilocybin trials, previous psychedelic use among participants, when reported, is much greater than in the general population (e.g. Carhart-Harris et al., 2021; Griffiths et al., 2016; Moreno et al., 2006). Explanations for these disproportionate numbers include self-referral to trials and some studies requiring past experiences. To extrapolate these ongoing findings to the general population, more diverse groups of participants should be recruited.

The efficacy of the blinding was not assessed in this study and therefore we cannot rule out the potential that guessing the treatment assignment influenced results. Participants underwent individual, not group, integration sessions after dosing in order to try to reduce unblinding. It is of note that four participants in the placebo arm did not complete the study. Given psilocybin’s psychedelic effects, combined with the fact that 37% of the total sample had previous experience using psilocybin, it is possible that these participants may have been able to determine their assigned treatment group.

It is possible that practice effects could have influenced results. However, a familiarisation session, where participants completed the cognitive assessments at the screening visit, was conducted. The purpose of familiarisation was to ensure that all the participants had a chance to practice the tests prior to the collection of the baseline data. This ensured that all participants understood the tests and minimised the small but consistent improvements in performance due to practice effects.

## Conclusions

This study demonstrated the feasibility of one-to-one psychological support from specially trained therapists during simultaneous administration of psilocybin in a supervised clinical setting in healthy volunteers. A single dose of psilocybin 10 mg or 25 mg elicited no serious AEs and did not appear to produce any clinically relevant detrimental short- or long-term effects, compared with placebo, in cognitive or social functioning or emotional regulation in this study in healthy volunteers. Further investigation of simultaneous therapeutic psilocybin administration in clinical populations is warranted.

## Supplemental Material

sj-docx-1-jop-10.1177_02698811211064720 – Supplemental material for The effects of psilocybin on cognitive and emotional functions in healthy participants: Results from a phase 1, randomised, placebo-controlled trial involving simultaneous psilocybin administration and preparation Click here for additional data file. Supplemental material, sj-docx-1-jop-10.1177_02698811211064720 for The effects of psilocybin on cognitive and emotional functions in healthy participants: Results from a phase 1, randomised, placebo-controlled trial involving simultaneous psilocybin administration and preparation by James J Rucker, Lindsey Marwood, Riikka-Liisa J Ajantaival, Catherine Bird, Hans Eriksson, John Harrison, Molly Lennard-Jones, Sunil Mistry, Francesco Saldarini, Susan Stansfield, Sara J Tai, Sam Williams, Neil Weston, Ekaterina Malievskaia and Allan H Young in Journal of Psychopharmacology
