# Supplementary material for: The effects of psilocybin on cognitive and emotional functions in healthy participants: Results from a phase 1, randomised, placebo-controlled trial involving simultaneous psilocybin administration and preparation
Source: J Psychopharmacol. 2022 Jan 4;36(1):114–25. doi: 10.1177/02698811211064720 (PMC8801675; doi:10.1177/02698811211064720)
Supplement: sj-docx-1-jop-10.1177_02698811211064720 – Supplemental material for The effects of psilocybin on cognitive and emotional functions in healthy participants: Results from a phase 1, randomised, placebo-controlled trial involving simultaneous psilocybin administration and preparation [file sj-docx-1-jop-10.1177_02698811211064720.docx]

# The effects of psilocybin on cognitive and emotional functions in healthy participants: results from a phase 1, randomised, placebo-controlled trial involving simultaneous psilocybin administration and preparation

James J Rucker^1,2^ (MD), Lindsey Marwood^3^ (PhD), Riikka-Liisa J Ajantaival^4^ (MA), Catherine Bird^1^ (MSc), Hans Eriksson^3^ (MD), John Harrison^1,6,7^ (PhD), Molly Lennard-Jones^3^ (MA), Sunil Mistry^3^ (MSc), Francesco Saldarini^3^ (MSc), Susan Stansfield^3^ (PhD), Sara J Tai^5^ (DClinPsy), Sam Williams^3^ (MSc), Neil Weston^1^ (MRCPsych), Ekaterina Malievskaia^3^ (MD), and Allan H Young^1,2^ (FRCPsych)

*^1^Department of Psychological Medicine, Institute of Psychiatry, Psychology & Neuroscience, King’s College London, London, UK; ^2^South London and Maudsley NHS Foundation Trust, London, UK; ^3^COMPASS Pathways plc, London, UK; ^4^Clinical Research Institute, Helsinki University Central Hospital, Helsinki, Finland; ^5^Division of Psychology and Mental Health, The University of Manchester, Manchester, UK; ^6^Alzheimer’s Center AUmc, Amsterdam, The Netherlands; ^7^Metis Cognition Ltd., Kilmington Common, UK*

**Correspondence:** James Rucker, The Institute of Psychiatry, Psychology and Neuroscience, King’s College London, 16 De Crespigny Park, London SE5 8AF, UK; Email: [james.rucker@kcl.ac.uk](mailto:james.rucker@kcl.ac.uk); Tel: +44 (0) 207 848 0088
